# RETRACTED: Caffo et al. Molecular Investigation of DKK3 in Cerebral Ischemic/Reperfusion Injury. *Biomedicines* 2023, *11*, 815

**DOI:** 10.3390/biomedicines14051016

**Published:** 2026-04-30

**Authors:** Maria Caffo, Roberta Fusco, Rosalba Siracusa, Gerardo Caruso, Valeria Barresi, Rosanna Di Paola, Salvatore Cuzzocrea, Antonino Francesco Germanò, Salvatore Massimo Cardali

**Affiliations:** 1 Department of Biomedical, Dental and Morphological and Functional Imaging, University of Messina, Via Consolare Valeria, 98125 Messina, Italy; 2 Department of Chemical, Biological, Pharmaceutical and Environmental Sciences, University of Messina, Viale Ferdinando Stagno D’Alcontres, n 31, 98166 Messina, Italy; 3 Department of Diagnostics and Public Health, University of Verona, Piazzale Ludovico Antonio Scuro, 37124 Verona, Italy; 4 Department of Veterinary Sciences, University of Messina, Viale Annunzita, 98168 Messina, Italy

The journal retracts the article titled “Molecular Investigation of DKK3 in Cerebral Ischemic/Reperfusion Injury” [1], cited above.

Following publication, concerns were brought to the attention of the Editorial Office regarding the integrity of a figure presented in this publication [1].

In accordance with the journal’s standard procedures, the Editorial Office and Editorial Board conducted an investigation that identified indications of inappropriate editing in Figure 5. Although the authors cooperated with the investigation, the explanations and supporting materials provided were not deemed sufficient by the Editorial Board to resolve the identified concerns. As a consequence, the Editorial Board has lost confidence in the reliability of the reported findings and has decided to retract this publication in accordance with MDPI’s retraction policy.

This retraction was approved by the Editor-in-Chief of the *Biomedicines* journal.

The authors have been informed of this retraction but did not provide a comment on this decision.
